# Intersectoral collaboration and healthcare for homeless people: an experience report

**DOI:** 10.1590/0034-7167-2025-0219

**Published:** 2026-10-19

**Authors:** Eduardo Sodré de Souza, Gabrielle Favotto Betoni, Débora Rodrigues Vaz, Chennyfer Dobbins Abi Rached

**Affiliations:** I Universidade Estadual de Campinas. Campinas, SP, Brazil; II Universidade de São Paulo. São Paulo, SP, Brazil

**Keywords:** Intersectoral Collaboration, Social Participation, Homeless Persons, Health Policy, Social Vulnerability., Colaboración Intersectorial, Participación Social, Personas Mal Alojadas, Política de Salud, Vulnerabilidad Social.

## Abstract

**Objective:**

to report the experience of using the World Café methodology, focusing on healthcare for the homeless population.

**Method:**

a qualitative experience report, with thematic analysis, centered on the implementation of the World Café methodology, carried out in the municipality of Suzano, SP, in November 2024. Ninety-two people from different sectors of the healthcare network, universities, and social movements participated.

**Results:**

the methodology proved effective in creating a horizontal and collaborative environment, promoting dialogue, active listening, and valuing diverse knowledge. Data analysis revealed three central topics: The methodology’s integrative potential; Structural challenges in caring for the homeless population; and local strategies and effective care practices.

**Final considerations:**

the experience indicates that participatory methodologies are powerful tools for training, planning, and social mobilization, with a positive impact on the construction of public policies sensitive to social vulnerabilities.

## RESUMEN

**Objetivo:**

informar sobre la experiencia de la metodología World Café, con énfasis en la atención sanitaria para la población sin hogar.

**Método:**

informe cualitativo de experiencia con análisis temático, centrado en la implementación de la metodología World Café en el municipio de Suzano, SP, en noviembre de 2024. Participaron noventa y dos personas de diferentes sectores de la Red de Atención Sanitaria, universidades y movimientos sociales.

**Resultados:**

la metodología demostró su eficacia para crear un entorno horizontal y colaborativo, fomentando el diálogo, la escucha activa y la valoración del conocimiento diverso. El análisis de datos reveló tres temas centrales: El potencial integrador de la metodología; los desafíos estructurales en la atención a la población sin hogar; y las estrategias locales y las prácticas de atención eficaces.

**Consideraciones finales:**

la experiencia demuestra que las metodologías participativas son herramientas poderosas para la capacitación, la planificación y la movilización social, con un impacto positivo en la formulación de políticas públicas sensibles a las vulnerabilidades sociales.

## INTRODUCTION

Characterized as a “heterogeneous population group that shares extreme poverty, interrupted or weakened family ties, and the lack of regular conventional housing”, the homeless population (HP) has increased significantly in Brazil. The number of people registered in the *Cadastro Único* between 2018 and 2023 almost doubled, reaching 221,113 Brazilians in this situation^(1)^.

A clear expression of an unequal and exclusionary society, homeless people, which in Brazil totals more than 236,400 people, distributed across more than 64% of the country’s municipalities, share a daily life marked by extreme poverty and precarious living and working conditions^(1)^.

However, while paying attention to this point, it is necessary to avoid the reductionism that homogenizes this population group, since their experiences are shaped by various social markers of difference, such as race/color, sex, gender, social class, generation, region, among others^(1)^. Added to this is the fragility of social and community networks, which highlights the complexity of caring for these individuals^(2)^.

Despite being constitutionally provided for and reinforced by the Brazilian National Policy for the Homeless Population^(1)^, regarding the right of the State to guarantee broad, simplified and secure access to the programs and services that comprise public policies, a great difficulty is observed for this population in accessing Brazilian Health System (In Portuguese, *Sistema Único de Saúde* -SUS) services.

Stigma and dehumanization, mediated by often inaccurate and distorted representations of these people’s reality, result in barriers to access to healthcare and, consequently, in worse outcomes when compared to the general population.

Aspects such as lack of documentation and proof of residence also constitute institutional barriers to this population’s access to health goods and resources, exacerbating the low adherence to the programs and actions offered. It is common for people to seek healthcare services only in extreme situations, when the worsening of their illness or distress restricts their activities of daily living.

Thus, interministerial initiatives such as Ordinance 1,127 of August 2015, from the Ministry of Health and the Ministry of Education, acknowledge the complexity of the homeless situation and propose coordinated strategies for comprehensive care. However, challenges persist^(1)^.

Overcoming these challenges requires recognizing the importance of strategies aimed at strengthening and sustaining network-based work, an essential condition for dealing with such complexity. The integration of different services and sectors of society to fill gaps in care for homelessness people demands intersectoral coordination, with effective involvement from public management and integration between service, education, and the community.

With a view to improving morbidity and mortality indicators in this population, the fundamental role of higher education institutions in offering teaching and learning strategies geared towards SUS demands is reinforced, aiming at professional qualification, especially with regard to overcoming the dichotomy between theory and practice.

The integration of different social actors in the planning, execution, and assessment of health actions aimed at the HP-including these individuals themselves as active participants in decision-making processes-constitutes a powerful path for the development of more qualified and socially committed care strategies. The collaboration between health and social assistance professionals, public managers, researchers, and community representatives promotes practices that are more sensitive to the vulnerabilities present in these territories. In this scenario, popular participation and intersectoral dialogue become essential foundations for building effective care networks and formulating public policies guided by equity, co-responsibility, and social justice.

## OBJECTIVE

To report on the experience of using the World *Café* methodology, focusing on healthcare for the HP.

## METHODS

This article presents an experience report on the application of the World *Café* active methodology as a formative and collective mobilization strategy aimed at critical reflection on healthcare practices for the HP in a municipality in the Metropolitan Region of São Paulo, on November 25, 2024, at the *Centro Universitário Piaget* campus, in Parque Suzano, Suzano, SP. The report seeks to describe, analyze, and reflect on the planning and implementation process, and the effects observed from the use of this methodology, in light of theoretical frameworks on participatory practices and meaningful learning. The EQUATOR checklist used to guide the study was the COnsolidated criteria for REporting Qualitative research tool.

The method described in this report was applied in a descriptive and interpretive research project based on the experience of a training event conducted by a university, public services, and social movements. The experience report format was chosen for its relevance in systematizing practices and producing situated knowledge. The intersectoral thematic seminar methodology was structured using the World *Café* technique, which, based on collective intelligence and horizontal dialogue, created welcoming spaces for conversation, encouraging several perspectives and collaborative construction of meaning^(1,3)^.

Planning the activity involved the participatory definition of the objectives, topics, and guiding questions, as well as the composition of an interprofessional team responsible for conducting the dialogue sessions, consisting of researchers/professors (4), nurses (4), social worker (1), psychologist (1), and nursing students (2). This group was responsible for the collective decision to size physical, human and material resources based on recommendations on the method^(3,4)^ and information shared with/by the Municipal Health Department, the research’s direct interlocutor.

Preparation involved a training workshop on the World *Café*, defining roles and logistical planning, considering available resources, and HP and service network participation. Execution prioritized welcoming and dialogue, with round tables, aesthetic ambiance, and accessible language, aligned with the principles of the methodology.

The event was attended by professionals from different sectors of the Healthcare Network and social assistance, including Street Clinic, Oral Health, Expanded Family Health Center, Basic Health Unit, Emergency Room, Social Assistance Reference Center, Specialized Social Assistance Reference Center, Psychosocial Care Center, as well as teachers, university students, and representatives of social movements from the HP, totaling 92 people, distributed between the morning (40) and afternoon (52) shifts. Inclusion criteria comprised being 18 years of age or older, having been invited by the research team or the Municipal Health Department, and voluntarily agreeing to participate in the study. Exclusion criterion comprised the identification of any cognitive or consciousness impairment, such as the effect of psychoactive substances, that could compromise individuals’ ability to participate.

This diverse group included individuals invited directly by both the research team (HP segment) and the Municipal Health Department, as well as professionals, public managers, and service providers. This integration and diversity of segments involved with the topic contributed significantly to the richness and complexity of the discussions, promoting a broader understanding and a dialogue between technical-scientific, popular, and institutional knowledge.

Participants were organized into small groups and circulated through different discussion tables over three conversational cycles, each guided by a pre-defined question: what are the challenges to healthcare for homelessness people? How do you care for homelessness people who use alcohol and drugs? And what are the scope and limitations of the service network that assists HP? Each table had a fixed host, responsible for recording the main points discussed and sharing the learnings with the new members in each round, and a mediator, who facilitated the flow and respectful listening within the group.

Instruments were used to collect sociodemographic data and information about the service from all participants. The discussions held at the thematic tables throughout the three conversational cycles were collaboratively and freely systematized on sheets of paper, with records kept by hosts and rapporteurs. Additionally, for each of the three guiding questions, specific standard forms were used for each table, totaling nine forms at the end of the event.

Each form was completed collaboratively by participants at the tables, with the support of facilitators, containing the main points discussed in each round, including challenges, strategies, and perceptions. Student observers also recorded free notes based on the groups’ interactions and responses, which allowed for data triangulation and deeper interpretation. The main findings of each group were presented in a final plenary session by the participants themselves, and observers’ notes were shared by a researcher.

All this material (papers, forms, and observer records) was consolidated through a preliminary reading and analyzed using inductive thematic analysis, with the aim of identifying discursive patterns, recurring challenges, and powerful care practices. The results were organized into interpretative axes that articulate the emerging content with World *Café*’s potential as an active teaching-learning methodology, intersectoral articulation, and situated knowledge production. This methodological strategy sought to ensure data reliability and transparency in the process of systematization and qualitative interpretation.

### Ethical aspects

The study was conducted in accordance with national and international ethical guidelines, and was approved by the Research Ethics Committee of the School of Nursing at the *Universidade de São Paulo*, whose opinion is attached to this submission.

## RESULTS

The analysis was based on the triangulation between the nine standard forms completed at the thematic tables; collaborative records were made on sheets of paper; and free-form notes were produced by student observers. The contributions were systematized into three thematic axes, according to the recurrence and density of the content emerging in dialogue sessions, totaling 75 records.

### Axis 1: World *Café*’s integrating potential

In this section, 17 records were analyzed. The application of the World *Café* methodology proved effective in creating a collective and horizontal learning environment conducive to dialogue, active listening, and the valuing of diverse knowledge. The rotation between tables allowed for the circulation of participants and the constant exchange of experiences, favoring the construction of a broader and interdisciplinary vision of healthcare (Chart 1).

**Chart 1:** Excerpts on the World *Café* methodology’s integrative potential. São Paulo, SP, Brazil

Participant	Quote
Expanded Family Health Center worker	“It was the first time I was able to speak directly with someone from the Street Clinic, and that opened my mind to other forms of care.”
Nursing student	“I realized that what we learn in college only makes sense when we listen to those who are in the field.”
Homeless population representative	“Here we didn’t just listen, we really had a voice, we were heard.”

### Axis 2: Structural challenges in providing care to the homeless population

The analysis of the 36 transcribed records revealed a series of structural challenges faced by teams and users in caring for the HP. Among the most frequently mentioned aspects are the network fragmentation, the scarcity of resources, the absence of specific protocols, and the lack of coordination between the health and social assistance sectors (Chart 2).

**Chart 2:** Excerpts on the challenges related to the care of people with disabilities. São Paulo, SP, Brazil

Participant	Quote
Basic Health Unit professional	“We assist many homelessness people, but we lack adequate training and sometimes don’t even know how to provide them with support.”
Psychosocial Care Center Worker	“The network is very fragile; it seems like each service is working in isolation.”
Health manager	“Specific policies are lacking; we’re just patching things up with what we have.”

### Axis 3: Local strategies and care practices

Despite the challenges, participants in 22 surveys also reported innovative local initiatives based on community ties, territoriality, and institutional creativity. These strategies point to concrete paths for promoting equitable care, even in adverse contexts (Chart 3).

**Chart 3:** Excerpts on local strategies and care practices. São Paulo, SP, Brazil

Participant	Quote
Street Clinic professional	“Often, the connection is what guarantees the person’s return to the service, more so than the scheduling.”
Social Assistance Reference Center technician	“When we manage to have a dialogue with the CAPS and the BHU, we are able to resolve many issues.”
Homeless population representative	“We take care of ourselves too. There are people on the street who help those who are unwell.”

**Summary: Analytical categories of thematic analysis (Chart 4**)

**Chart 4:** Emerging analytical categories in thematic analysis. São Paulo, SP, Brazil

Thematic axis	Analytical categories	Related subtopics
Integrative potential	Horizontal nature of dialogue	Active listening, exchange of knowledge, integration of teaching and service
Structural challenges	Network’s fragility	Lack of coordination, scarce resources, absence of protocols
Powerful practices	Strategies for connection and territoriality	Relationship as care, informal networks, effective intersectorality

## DISCUSSION

Intersectoral collaboration, mediated by participatory methodological strategies, demonstrated its potential in consolidating public policies on HP’s health, highlighting the integration of different segments of society: community, universities, services, and public management. The challenges and potentials that emerged in this process inform these different segments about the limits, possibilities, and challenges inherent in health policies and practices for homeless people. These elements will be discussed below.

### Axis 1: World *Café*’s integrating potential

The findings related to this axis demonstrate that the participatory approach of the used methodology confirms its role in fostering connections among various social actors and facilitating the co-creation of knowledge applicable to the local context^(3,4)^.

“World *Café*’s integrative potential: dialogue as a formative and political practice” allowed for a break from traditional training models centered on the vertical transmission of knowledge. Furthermore, a dialogical, horizontal, and collaborative space was created, in which different social actors, such as workers, students, managers, and HP representatives, were able to express their experiences, construct shared meanings, and identify points of convergence and conflict in care practice.

Active listening, respect for the plurality of knowledge, and circularity in discussion groups contributed to mutual recognition among professionals from different sectors, promoting interdisciplinarity and strengthening network cohesion. This characteristic aligns with the ideas of Paulo Freire, who believed that dialogue is a cognitive and political act that transforms individuals by allowing them to recognize themselves as protagonists of their own reality^(5)^.

Educational spaces that promote critical dialogue contribute to restoring the ethical-political dimension of care, especially when articulated with concrete social demands, such as those experienced by the HP^(6)^. In this regard, the World *Café* was not limited to a facilitation technique, but operated as a pedagogy of listening and co-responsibility, promoting situated learning committed to social transformation.

### Axis 2: Structural challenges in providing care to the homeless population

The statements categorized in this section reflect the mismatch between the normative principles of the SUS and the actual working conditions in its services. Recognizing these obstacles is essential to guide structural changes and strengthen intersectoral collaboration.

The structural challenges in caring for the HP reveal a fragmented system. Systematized accounts from discussion groups point to persistent obstacles that compromise the effectiveness of care, such as care network disarticulation, scarcity of resources, absence of specific protocols, and limited intersectoral coordination-recurring aspects that corroborate the literature on the precariousness of healthcare for highly vulnerable populations^(7)^.

These problems reveal a dissonance among the SUS principles - universality, comprehensiveness, and equity - and its implementation in the territories. The absence of coordinated care, high staff turnover, low training levels for people caring for those with HIV/AIDS, and the institutional invisibility of this group reveal that the care model is still based on biomedical and fragmented logics.

It is understood that this fragmentation stems, to a large extent, from bureaucratic rigidity and the lack of effective mechanisms for participatory management^(8)^. Listening to workers and users revealed that actions aimed at promoting sexual and reproductive health often function exceptionally, depending on individual goodwill or informal arrangements between services, which highlights the deinstitutionalization of public policies aimed at equity.

### Axis 3: Local strategies and care practices

The analyzed experiences reinforce the importance of valuing territorial knowledge and recognizing individuals as active agents in care. Even in adverse contexts, powerful, creative, and emotional local initiatives based on connection, listening, and territoriality emerged. The Street Clinic exemplifies ethical, sensitive, and continuous care aligned with expanded clinical practice that values its users’ uniqueness^(9)^.

Informal care networks, formed by professionals and homelessness people, also became evident. These networks act as support structures and a means of resistance to the limitations of formal services, ensuring shelter and access to resources. These practices indicate the need for public policies that support local knowledge and break with decontextualized normative models. Co-management and institutional support are essential to sustain effective and humanized practices^(10)^.

The power of the active methodology used lay not only in the content, but in the collective production of knowledge. The World *Café* demonstrated that including the HP in policy formulation and assessment processes is fundamental to promoting more effective, democratic, and equitable actions, strengthening social empowerment and the construction of public policies aligned with this population’s real needs.

### Study limitations

The study limitations include its nature as an experience report, which highlighted intersectoral collaboration in providing healthcare to homeless individuals, making it impossible to generalize the findings, compare them, or validate their causality.

### Contributions to nursing, health, or public health

The article highlights the participatory World *Café* methodology’s potential in promoting intersectoral dialogue, critical professional training, and the development of more integrated and humanized care strategies by reporting on its application in the context of HP care.

This experience broadens our understanding of the importance of dialogical training practices that value local knowledge, active listening, and the participation of those involved, including the HP itself. These practices are essential for strengthening care networks and overcoming structural barriers to healthcare access.

Furthermore, this report presents a replicable methodological strategy that can be adapted to different territorial realities and used as an instrument for participatory planning and social mobilization. By integrating teaching, service, municipal management, and the community, the study emphasizes the importance of the relationship between universities and society in producing knowledge that is committed to social transformation.

## FINAL CONSIDERATIONS

The World *Café* experience reveals its potential as a political and pedagogical tool that goes beyond simple facilitation methodology. By promoting active listening, horizontal dialogue, and collective construction, the World *Café* enables the exchange of diverse knowledge, fostering critical learning, networking, and the formulation of intervention proposals in territories.

This approach broadened participants’ training and strengthened bonds among workers, managers, users, and researchers, contributing to public policies that are more sensitive to local realities and guided by social justice and health equity.

The importance of integrating education, service, and community is emphasized to promote shared responsibility and actions that better align with the HP’s needs. Including historically marginalized individuals in decision-making spaces is essential for more democratic and just practices.

There is a growing need to incorporate participatory methodologies, such as World *Café*, discussion groups, and intersectoral forums, into the processes of formulating and assessing public policies. These strategies are more than just technical tools; they restore the democratic dimension of social policies. Finally, it is recommended that these experiences be systematized and replicated to strengthen social leading role and commitment to equity and the right to a dignified life.

## Data Availability

The available research data are in the body of the article.
