# Supplementary material for: Intersectoral collaboration and healthcare for homeless people: an experience report
Source: Rev Bras Enferm. 2026 Oct 19;79:e20250219. doi: 10.1590/0034-7167-2025-0219 (PMC13679587; doi:10.1590/0034-7167-2025-0219)
Supplement: Translation

# Translation: Colaboração intersetorial e atenção à saúde de pessoas em situação de rua: relato de experiência

**DOI:** 10.1590/0034-7167-2025-0219pt

**Authors:** Eduardo Sodré de Souza, Gabrielle Favotto Betoni, Débora Rodrigues Vaz, Chennyfer Dobbins Abi Rached

**Affiliations:** I Universidade Estadual de Campinas. Campinas, SP, Brasil; II Universidade de São Paulo. São Paulo, SP, Brasil

**Keywords:** Colaboração Intersetorial, Participação Social, Pessoas Mal Alojadas, Política de Saúde, Vulnerabilidade Social.

## Abstract

**Objetivo:**

relatar a experiência do uso da metodologia *World* Café, com foco na atenção à saúde da população em situação de rua.

**Método:**

relato de experiência de abordagem qualitativa, com análise temática, centrado na realização da metodologia *World* Café, realizada no município de Suzano, SP, em novembro de 2024. Participaram 92 pessoas de diferentes setores da Rede de Atenção à Saúde, universidades e movimentos sociais.

**Resultados:**

a metodologia demonstrou-se eficaz na criação de ambiente horizontal e colaborativo, promovendo diálogo, escuta ativa e valorização de saberes diversos. A análise de dados revelou três eixos centrais: O potencial integrador da metodologia; Os desafios estruturais no cuidado à população em situação de rua; e As estratégias locais e práticas potentes de cuidado.

**Considerações finais:**

a experiência aponta que metodologias participativas são potentes ferramentas de formação, planejamento e mobilização social, com impacto positivo na construção de políticas públicas sensíveis às vulnerabilidades sociais.

## INTRODUÇÃO

Caracterizada como “grupo populacional heterogêneo que possui em comum a pobreza extrema, os vínculos familiares interrompidos ou fragilizados e a inexistência de moradia convencional regular”, a população em situação de rua (PSR) tem aumentado significativamente no Brasil. O número de pessoas cadastradas no Cadastro Único entre 2018 e 2023 quase dobrou, sendo 221.113 brasileiros nessa condição^(1)^.

Expressão clara de uma sociedade desigual e excludente, as pessoas em situação de rua, que somam no Brasil mais de 236.400 pessoas, distribuídas em mais de 64% dos municípios do país, compartilham o cotidiano marcado pela pobreza extrema e pela precarização da vida e do trabalho^(1)^.

Com atenção a este ponto, é preciso, no entanto, evitar o reducionismo que homogeneíza esse grupo populacional, uma vez que suas vivências e experiências são atravessadas por diversos marcadores sociais da diferença, como raça/cor, sexo, gênero, classe social, geração, região, entre outros^(1)^. Soma-se a isso a fragilidade das redes sociais e comunitárias, que expressa a complexidade do cuidado para essas pessoas^(2)^.

Apesar de constitucionalmente previsto e reforçado pela Política Nacional para a População em Situação de Rua^(1)^, em relação ao direito de que o Estado garanta o acesso garanta o acesso amplo, simplificado e seguro aos programas e serviços que compõem as políticas públicas, observa-se uma grande dificuldade dessa população em acessar os serviços do Sistema Único de Saúde (SUS).

O estigma e a desumanização, mediados por representações muitas vezes equivocadas e distorcidas sobre a realidade dessas pessoas, resultam em barreiras ao acesso à saúde e, consequentemente, em piores desfechos, quando comparados à população em geral.

Aspectos como ausência de documentação e de comprovante de residência também se configuram como barreiras institucionais ao acesso dessa população aos bens e recursos da saúde, agravando a baixa adesão aos programas e ações ofertados. É comum a busca por serviços de saúde apenas em situações-limite, quando a agudização do quadro de adoecimento ou sofrimento restringe suas atividades de vida diária.

Dessa forma, iniciativas interministeriais como a Portaria nº 1.127, de agosto de 2015, do Ministério da Saúde e do Ministério da Educação, reconhecem a complexidade da situação de rua e propõem estratégias articuladas para uma atenção integral. Entretanto, os desafios persistem^(1)^.

A superação desses desafios passa pelo reconhecimento da importância de estratégias voltadas ao fortalecimento e à sustentabilidade do trabalho em rede, condição essencial para lidar com tamanha complexidade. A integração entre diferentes serviços e setores da sociedade, para o preenchimento das lacunas no cuidado às pessoas em situação de rua, exige articulação intersetorial, com envolvimento efetivo da gestão pública e integração entre serviço, ensino e comunidade.

Com vistas à melhoria dos indicadores de morbimortalidade dessa população, reforça-se o papel fundamental das instituições de ensino superior na oferta de estratégias de ensino-aprendizagem voltadas às demandas do SUS, visando à qualificação profissional, especialmente no que se refere à superação da dicotomia entre teoria e prática.

A integração de diferentes atores e atrizes sociais no planejamento, execução e avaliação de ações em saúde voltadas à PSR - incluindo essas próprias pessoas como participantes ativos dos processos decisórios - constitui um caminho potente para o desenvolvimento de estratégias de cuidado mais qualificadas e socialmente comprometidas. A articulação entre profissionais de saúde e de assistência social, gestores públicos, pesquisadores, e representantes da comunidade promove práticas mais sensíveis às vulnerabilidades presentes nos territórios. Nesse cenário, a participação popular e o diálogo intersetorial se tornam fundamentos essenciais para construção de redes de atenção efetivas e formulação de políticas públicas orientadas pela equidade, corresponsabilidade e justiça social.

## OBJETIVO

Relatar a experiência do uso da metodologia *World* Café, com foco na atenção à saúde da PSR.

## MÉTODOS

Este artigo apresenta um relato de experiência sobre a aplicação da metodologia ativa *World* Café como estratégia formativa e de mobilização coletiva voltada à reflexão crítica sobre práticas de cuidado em saúde da PSR em um município da Região Metropolitana de São Paulo, em 25 de novembro de 2024, no *campus* do Centro Universitário Piaget, no Parque Suzano, Suzano, SP. O relato busca descrever, analisar e refletir sobre o processo de planejamento, implementação e os efeitos observados a partir do uso dessa metodologia, à luz de referenciais teóricos sobre práticas participativas e aprendizagem significativa. A lista de verificação EQUATOR usada para nortear o estudo foi a ferramenta *COnsolidated criteria for REporting Qualitative research*.

O método objeto deste relato foi aplicado em pesquisa, de caráter descritivo e interpretativo, baseada na experiência de evento formativo realizado por universidade, serviços públicos e movimentos sociais. Optou-se pelo relato de experiência por sua relevância na sistematização de práticas e na produção de conhecimento situado. A metodologia do seminário temático intersetorial foi estruturada com a técnica *World* Café, que, baseada na inteligência coletiva e no diálogo horizontal, criou espaços de conversa acolhedores, estimulando múltiplas perspectivas e a construção colaborativa de sentidos^(1,3)^.

O planejamento da atividade envolveu a definição participativa dos objetivos, temas e perguntas norteadoras, assim como a composição de equipe interprofissional responsável por conduzir as rodas de diálogo, sendo pesquisadores/professores da universidade (4), enfermeiros (4), assistente social (1), psicólogo (1) e estudantes de enfermagem (2). A este grupo coube a decisão coletiva de dimensionar recursos físicos, humanos e materiais com base nas recomendações sobre o método^(3,4)^ e informações compartilhadas com/pela a Secretaria Municipal de Saúde, interlocutora direta da pesquisa.

A preparação envolveu oficina formativa sobre o *World* Café, definição de papéis e planejamento logístico, considerando recursos disponíveis e participação da PSR e da rede de serviços. A execução priorizou acolhimento e diálogo, com mesas em círculo, ambientação estética e linguagem acessível alinhada aos princípios da metodologia.

Participaram do evento profissionais de diferentes setores da Rede de Atenção à Saúde e da assistência social, incluindo Consultório na Rua, Saúde Bucal, Núcleo Ampliado de Saúde da Família, Unidade Básica de Saúde, Pronto-Socorro, Centro de Referência da Assistência Social, Centro de Referência Especializado de Assistência Social, Centro de Atenção Psicossocial, além de docentes, estudantes universitários e representantes de movimentos sociais da PSR, totalizando 92 pessoas, distribuídas entre os turnos matutino (40) e vespertino (52). Os critérios de inclusão para o estudo foram ter 18 anos ou mais, ter sido convidado pela equipe de pesquisa ou pela Secretaria Municipal da Saúde e aceitar participar voluntariamente do estudo. O critério de exclusão adotado foi a identificação de qualquer comprometimento cognitivo ou de consciência, como o efeito de substâncias psicoativas, que pudesse comprometer a capacidade do indivíduo de participar.

Essa composição plural contou com convites feitos diretamente tanto pela equipe de pesquisa (segmento de PSR) quanto pela Secretaria Municipal da Saúde, profissionais, além de gestores públicos e de serviços. Essa integração e diversidade de segmentos envolvidos com a temática contribuíram significativamente para a riqueza e complexidade das discussões, promovendo uma escuta ampliada e um diálogo entre saberes técnico-científicos, populares e institucionais.

Os participantes foram organizados em pequenos grupos, circulando por diferentes mesas de discussão ao longo de três ciclos conversacionais, cada qual guiado por uma pergunta previamente definida, a saber: quais os desafios ao cuidado à saúde das pessoas em situação de rua? Como vocês cuidam das pessoas em situação de rua que fazem o uso de álcool e drogas? E quais os alcances e limites da rede de serviços que atende às PSR? Cada mesa contou com um anfitrião fixo, responsável por registrar os principais pontos discutidos e compartilhar os aprendizados com os novos integrantes em cada rodada, e com um mediador, que facilitava a fluidez e o respeito à escuta no grupo.

Foram aplicados instrumentos para coleta de dados sociodemográficos e informações sobre o serviço para todos os participantes. As discussões realizadas nas mesas temáticas ao longo dos três ciclos conversacionais foram sistematizadas, de forma colaborativa e livre, em folhas de sulfite, com registros conduzidos por anfitriões e relatores. Complementarmente, para cada uma das três perguntas norteadoras, foram utilizados formulários-padrão específicos por mesa, totalizando nove formulários ao final do evento.

Cada formulário foi preenchido de forma conjunta pelos participantes das mesas, com apoio dos facilitadores, contendo os principais pontos debatidos em cada rodada, incluindo desafios, estratégias e percepções. Estudantes observadores também registraram anotações livres com base nas interações e respostas dos grupos, o que possibilitou a triangulação dos dados e o aprofundamento das interpretações. Os principais achados de cada grupo foram apresentados em plenária final pelos próprios participantes, e as anotações dos observadores foram compartilhadas por um pesquisador.

Todo esse material (sulfites, formulários e registros dos observadores) foi consolidado por meio de leitura flutuante e analisado por meio de análise temática indutiva, com o objetivo de identificar padrões discursivos, desafios recorrentes e práticas potentes de cuidado. Os resultados foram organizados em eixos interpretativos que articulam os conteúdos emergentes às potencialidades do *World* Café como metodologia ativa de ensino-aprendizagem, articulação intersetorial e produção de conhecimento situado. Essa estratégia metodológica buscou garantir a fidedignidade dos dados e a transparência no processo de sistematização e interpretação qualitativa.

### Aspectos éticos

O estudo foi conduzido de acordo com as diretrizes de ética nacionais e internacionais, e aprovado pelo Comitê de Ética em Pesquisa da Escola de Enfermagem da Universidade de São Paulo, cujo parecer está anexado à presente submissão.

## RESULTADOS

A análise foi baseada na triangulação entre os nove formulários-padrão preenchidos nas mesas temáticas; os registros colaborativos foram feitos em folhas de sulfite; e as anotações livres foram produzidas por estudantes observadores. As contribuições foram sistematizadas em três eixos temáticos, de acordo com a recorrência e densidade dos conteúdos emergentes nas rodas de diálogo, totalizando 75 registros.

### Eixo 1: Potencial integrador do *World* Café

Neste eixo, foram analisados 17 registros. A aplicação da metodologia *World* Café se mostrou eficaz na criação de um ambiente de aprendizagem coletivo e horizontal propício ao diálogo, à escuta ativa e à valorização dos saberes diversos. O rodízio entre as mesas permitiu a circulação dos participantes e a troca constante de experiências, favorecendo a construção de uma visão ampliada e interdisciplinar do cuidado em saúde (Quadro 1).

**Quadro 1:** Excertos sobre o potencial integrador da metodologia do *World* Café. São Paulo, SP, Brasil

Participante	Citação
Trabalhadora do Núcleo Ampliado de Saúde da Família	“Foi a primeira vez que pude conversar diretamente com alguém do Consultório na Rua, e isso abriu minha cabeça para outras formas de cuidado.”
Estudante de enfermagem	“Pude perceber que o que aprendemos na faculdade só faz sentido quando ouvimos quem está no território.”
Representante da PSR	“Aqui a gente não ficou só ouvindo, teve voz mesmo, foi escutado.”

### Eixo 2: Desafios estruturais no cuidado à população em situação de rua

A análise dos 36 registros transcritos evidenciou uma série de desafios estruturais enfrentados pelas equipes e pelos usuários no cuidado à PSR. Entre os aspectos mais mencionados, estão a fragmentação da rede, a escassez de recursos, a ausência de protocolos específicos, e a falta de articulação entre os setores de saúde e assistência social (Quadro 2).

**Quadro 2:** Excertos sobre os desafios relacionados ao cuidado à população em situação de rua. São Paulo, SP, Brasil

Participante	Citação
Profissional da Unidade Básica de Saúde	“Atendemos muitas pessoas em situação de rua, mas não temos formação adequada e às vezes nem sabemos como acolher.”
Trabalhadora do Centro de Atenção Psicossocial	“A rede é muito frágil, parece que cada serviço trabalha sozinho.”
Gestor da saúde	“Faltam políticas específicas, ficamos remendando com o que temos.”

### Eixo 3: Estratégias locais e práticas de cuidado

Apesar das dificuldades, os participantes em 22 registros também relataram iniciativas locais inovadoras baseadas em vínculos, territorialidade e criatividade institucional. Essas estratégias apontam caminhos concretos para promoção do cuidado com equidade, mesmo em contextos adversos (Quadro 3).

**Quadro 3:** Excertos sobre estratégias locais e práticas de cuidado. São Paulo, SP, Brasil

Participante	Citação
Profissional do Consultório na Rua	“Muitas vezes, o vínculo é o que garante o retorno da pessoa ao serviço, mais do que o agendamento.”
Técnica do Centro de Referência da Assistência Social	“Quando conseguimos dialogar com o CAPS e com a UBS, conseguimos resolver muita coisa.”
Representante da população em situação de rua	“A gente se cuida também. Tem gente na rua que ajuda quem está mal.”

**Síntese: Categorias analíticas da análise temática (Quadro 4)**

**Quadro 4:** Categorias analíticas emergentes da análise temática. São Paulo, SP, Brasil

Eixo temático	Categorias analíticas	Subtemas relacionados
Potencial integrador	Horizontalidade do diálogo	Escuta ativa, circulação entre saberes, integração ensino-serviço
Desafios estruturais	Fragilidade da rede	Falta de articulação, recursos escassos, ausência de protocolos
Práticas potentes	Estratégias de vínculo e territorialidade	Vínculo como cuidado, redes informais, intersetorialidade efetiva

## DISCUSSÃO

A colaboração intersetorial, mediada por estratégias metodológicas participativas, evidenciou o seu potencial na consolidação de políticas públicas sobre saúde da PSR, destacando-se a integração de diferentes segmentos da sociedade: comunidade, universidades, serviços e gestão pública. Os desafios e potenciais que emergiram neste processo informam esses diferentes segmentos sobre limites, possibilidades e desafios inerentes às políticas e práticas de saúde de PSR. A seguir, serão discutidos esses elementos.

### Eixo 1: Potencial integrador do *World* Café

Os achados relacionados a este eixo demonstram que a construção participativa proporcionada pela metodologia utilizada confirma seu papel na promoção de conexões entre diferentes atores sociais e na cocriação de saberes aplicáveis à realidade local^(3,4)^.

“O potencial integrador do *World* Café: diálogo como prática formativa e política” permitiu romper com modelos tradicionais de formação centrados na transmissão vertical do conhecimento. Além disso, produziu-se um espaço dialógico, horizontal e colaborativo, em que diferentes atores sociais, como trabalhadores, estudantes, gestores e representantes da PSR, puderam expressar suas vivências, construir sentidos compartilhados, e identificar pontos de convergência e conflito na prática do cuidado.

A escuta ativa, o respeito à pluralidade de saberes e a circularidade nas rodas de conversa contribuíram para o reconhecimento mútuo entre profissionais de diferentes setores, promovendo a interdisciplinaridade e fortalecendo a coesão da rede. Essa característica dialoga com Paulo Freire, para quem o diálogo é, ao mesmo tempo, um ato cognitivo e político, pois transforma os sujeitos, ao permitir que se reconheçam como protagonistas de sua realidade^(5)^.

Espaços de formação que promovem o diálogo crítico contribuem para resgatar a dimensão ético-política do cuidado, especialmente quando articulados a demandas sociais concretas, como as vividas pela PSR^(6)^. Nesse sentido, o *World* Café não se limitou a uma técnica de facilitação, mas operou como uma pedagogia da escuta e da corresponsabilização, promovendo aprendizagens situadas e comprometidas com a transformação social.

### Eixo 2: Desafios estruturais no cuidado à população em situação de rua

As falas categorizadas neste eixo refletem o descompasso entre os princípios normativos do SUS e as condições reais de trabalho nos serviços. O reconhecimento desses entraves é essencial para orientar mudanças estruturais e fortalecer a articulação intersetorial.

Os desafios estruturais no cuidado à PSR evidenciam um sistema fragmentado. Os relatos sistematizados nas rodas de conversa apontam obstáculos persistentes que comprometem a efetividade do cuidado, como a desarticulação da rede de atenção, a escassez de recursos, a ausência de protocolos específicos e a limitada articulação intersetorial - aspectos recorrentes que corroboram a literatura sobre a precariedade da atenção à saúde de populações em situação de alta vulnerabilidade^(7)^.

Esses problemas revelam uma dissonância entre os princípios do SUS - universalidade, integralidade e equidade - e sua implementação nos territórios. A ausência de fluxos coordenados, a alta rotatividade de profissionais, a baixa capacitação para o atendimento à PSR e a invisibilidade institucional desse grupo expõem um modelo de atenção ainda baseado em lógicas biomédicas e segmentadas.

Compreende-se que essa fragmentação decorre, em grande medida, da rigidez burocrática e da falta de mecanismos eficazes de gestão participativa^(8)^. A escuta de trabalhadores e usuários revelou que as ações voltadas à PSR, muitas vezes, funcionam de forma excepcional, dependendo da boa vontade individual ou de articulações informais entre os serviços, o que evidencia a desinstitucionalização das políticas públicas voltadas à equidade.

### Eixo 3: Estratégias locais e práticas potentes

As experiências analisadas reforçam a importância de valorizar os saberes do território e reconhecer os sujeitos como agentes ativos no cuidado. Mesmo em contextos adversos, emergiram iniciativas locais potentes, criativas e afetivas baseadas no vínculo, na escuta e na territorialidade. O Consultório na Rua se destacou como exemplo de cuidado ético, sensível e contínuo, alinhado à clínica ampliada, que valoriza a singularidade dos usuários^(9)^.

Também se evidenciaram as redes informais de cuidado, formadas por profissionais e por pessoas em situação de rua, que atuam como estruturas de apoio e resistência às limitações dos serviços formais, garantindo acolhimento e acesso a recursos. Essas práticas indicam a necessidade de políticas públicas que apoiem os saberes locais e rompam com modelos normativos descontextualizados. A cogestão e o apoio institucional são essenciais para sustentar práticas resolutivas e humanizadas^(10)^.

A potência da metodologia ativa utilizada residiu não apenas no conteúdo, mas na produção coletiva do conhecimento. O *World* Café demonstrou que incluir a PSR nos processos de formulação e avaliação de políticas é fundamental para promover ações mais eficazes, democráticas e equitativas, fortalecendo o protagonismo social e a construção de políticas públicas alinhadas às reais necessidades dessa população.

### Limitações do estudo

As limitações deste trabalho incluem a sua natureza de relato de experiência, que evidenciou a colaboração intersetorial para atenção à saúde de pessoas em situação de rua, não sendo possível a generalização dos achados, sua comparação ou validação causal.

### Contribuições para as áreas da enfermagem, saúde ou saúdes públicas

Ao relatar a aplicação da metodologia participativa *World* Café no contexto do cuidado à PSR, o artigo evidencia o potencial dessa abordagem na promoção do diálogo intersetorial, na formação crítica de profissionais, e na construção de estratégias de cuidado mais integradas e humanizadas.

A experiência apresentada amplia a compreensão sobre a importância de práticas formativas dialógicas que valorizem o saber local, a escuta ativa e a participação dos sujeitos envolvidos incluindo a própria PSR. Tais práticas se mostram fundamentais para o fortalecimento de redes de atenção e para a superação de barreiras estruturais no acesso à saúde.

Além disso, este relato apresenta uma estratégia metodológica replicável, que pode ser adaptada a diferentes realidades territoriais como instrumento de planejamento participativo e mobilização social. Ao integrar ensino, serviço, gestão municipal e comunidade, o estudo reforça a relevância da articulação entre universidade e sociedade na produção de conhecimento situado e comprometido com a transformação social.

## CONSIDERAÇÕES FINAIS

A experiência com o *World* Café revela seu potencial como dispositivo político e pedagógico, indo além de uma simples metodologia de facilitação. Ao promover escuta ativa, diálogo horizontal e construção coletiva, possibilitou a troca entre saberes diversos, fomentando a aprendizagem crítica, articulação em rede e formulação de propostas de intervenção nos territórios.

Essa abordagem ampliou a formação dos participantes, e fortaleceu os vínculos entre trabalhadores, gestores, usuários e pesquisadores, contribuindo para políticas públicas mais sensíveis às realidades locais e orientadas pela justiça social e equidade em saúde.

Destaca-se a importância da integração entre ensino, serviço e comunidade, promovendo corresponsabilização e ações mais aderentes às demandas da PSR. A inclusão de sujeitos historicamente marginalizados nos espaços de decisão se mostrou essencial para práticas mais democráticas e justas.

Reforça-se a necessidade de incorporar metodologias participativas, como o *World* Café, rodas de conversa e fóruns intersetoriais, aos processos de formulação e avaliação de políticas públicas. Mais que ferramentas técnicas, essas estratégias resgatam a dimensão democrática das políticas sociais. Recomendam-se, por fim, a sistematização e replicação dessas experiências, fortalecendo o protagonismo social e o compromisso com a equidade e o direito à vida digna.

## Data Availability

Os dados de pesquisa disponíveis estão no corpo do artigo.
